# CsPOM1, a DYRK Family Kinase, Plays Diverse Roles in Fungal Development, Virulence, and Stress Tolerance in the Anthracnose Pathogen *Colletotrichum scovillei*

**DOI:** 10.3389/fcimb.2022.861915

**Published:** 2022-04-26

**Authors:** Jong-Hwan Shin, Hee-Yeong Kim, Teng Fu, Kwang-Ho Lee, Kyoung Su Kim

**Affiliations:** Division of Bio-Resource Sciences, Bio-Herb Research Institute, and Interdisciplinary Program in Smart Agriculture, Kangwon National University, Chuncheon, South Korea

**Keywords:** cell division, DYRK, *Colletotrichum scovillei*, pepper anthracnose, Pom1

## Abstract

*Colletotrichum scovillei* is the major anthracnose fungus of sweet pepper and chili pepper (*Capsicum annuum* L.), causing significant losses in the yield and quality of the pepper fruits. Molecular mechanisms governing development and pathogenicity have been widely studied in many foliar fungal pathogens, but the information on fruit diseases is still limited. In this study, we determined the functional roles of the dual-specificity tyrosine phosphorylation-regulated kinase CsPOM1 in *C. scovillei*. Knockout mutant for *CsPOM1* gene was obtained *via* homology-dependent gene replacement. The *ΔCspom1* mutant exhibited a reduction in vegetative growth on osmotic stress, surface hydrophobicity, and conidiation compared with wild-type. Conidia of the *ΔCspom1* mutant were already two-celled before inoculation on an induction surface, indicating that *CsPOM1* negatively regulates conidial cell division. The *ΔCspom1* mutant, similar to wild-type, formed appressoria on the plant surface, but was significantly reduced on hydrophobic coverslips, probably due to a defect in the recognition of surface hydrophobicity. Treatment of conidia with cutin monomers restored appressorium formation on hydrophobic coverslips in the *ΔCspom1* mutant. On pepper fruits, the *ΔCspom1* mutant exhibited delayed penetration and invasive growth, leading to significantly reduced virulence. Collectively, the results showed that *CsPOM1* is important for stress tolerance, conidiation, surface hydrophobicity, appressorium formation, and virulence in *C. scovillei*.

## Introduction

The molecular mechanisms governing fungal development and pathogenicity of many foliar fungal pathogens have been extensively studied through functional genomics (Asakura et al., 2009; Wilson and Talbot, 2009). However, compared with foliar diseases, there is still limited information on fruit diseases, such as anthracnose caused by *Colletotrichum* species. The genus *Colletotrichum* is a major group of phytopathogenic fungi in the filamentous ascomycetes, causing anthracnose disease in many vegetables and fruits. The number of species within the genus ranges from 29 to more than 700, depending on taxonomic interpretation (Cannon et al., 2007; Dean et al., 2012). Due to the wide host range of *Colletotrichum* species, which included important crops such as apple, banana, coffeeberry, mango, and pepper, the genus is considered the eighth most important group of phytopathogenic fungi in the world (Dean et al., 2012). Anthracnose of sweet pepper and chili pepper (*Capsicum annuum* L.) caused by several *Colletotrichum* species, including *C*. *scovillei*, *C. truncatum*, *C. fructicola*, *C. dematium*, *C*. *gloeosporioides*, *C. coccodes*, and *C*. *capsici*, leads to significant losses in the yield and quality of pepper fruits in both pre- and postharvest periods (Caires et al., 2014; Kanto et al., 2014; Liu et al., 2016; Zhao et al., 2016; Diao et al., 2017). Recent advances in sequencing technology have revealed that *C. scovillei*, which belongs to the *C. acutatum* species complex is the dominant fungal pathogen of pepper anthracnose disease worldwide (Caires et al., 2014; Zhao et al., 2016; Diao et al., 2017; Noor and Zakaria, 2018; Khalimi et al., 2019). As *C. scovillei* was shown to be the most dominant fungal pathogen in pepper anthracnose in many countries, whole-genome sequencing, annotation, and genetic transformation methods for this pathogen have been established to understand fungal biology and pathogenicity (Han et al., 2016; Shin et al., 2019).

Fungi possess conserved signal transduction pathways, such as mitogen-activated protein kinase (MAPK) signaling pathways and the cyclic adenosine monophosphate (cAMP)-dependent protein kinase signaling pathway, which regulates developmental processes, stress responses, and virulence in many fungi (Xu and Hamer, 1996; Adachi and Hamer, 1998; Takano et al., 2000; Hagiwara et al., 2009; Mehrabi et al., 2009). Studying the mechanisms of fungal signaling systems provides information important for understanding fungal development and pathogenicity. One of the best-studied MAPK pathways is the Pmk1 MAPK pathway, which is required for appressorium formation and plant infection in many phytopathogenic fungi (Xu and Hamer, 1996; Zhao et al., 2007; Liang et al., 2019; Fu et al., 2022). The high osmolarity glycerol (HOG) MAPK pathway is also one of the well-characterized MAPK pathways that mediate cellular adaptation to hyperosmotic stress (Van Wuytswinkel et al., 2000; Tatebayashi et al., 2006; Wu et al., 2006; Hohmann, 2009; Duran et al., 2010; Saito and Posas, 2012). Recently, it has been revealed that a DYRK-family kinase PomA (a homolog of Pom1) is involved in the negative regulation of the HOG MAPK pathway in *Aspergillus nidulans*, affecting the phosphorylation level of Hog1, suggests important roles for PomA in the regulation of HOG MAPK pathway (Zhou et al., 2019).

DYRKs are serine/threonine protein kinases that autophosphorylate a tyrosine residue in their activation loop and phosphorylate their substrates on serine and threonine residues (Kentrup et al., 1996; Becker et al., 1998). The DYRK family can be classified into three subfamilies, i.e., DYRK kinases, homeodomain-interacting protein kinases, and pre-mRNA processing protein 4 kinases, based on the homology within the kinase domain (Aranda et al., 2011). Among them, Yak1 and Pom1 are well-characterized DYRK kinases in yeast (Garrett et al., 1991; Bähler and Pringle, 1998). Yak1, the first DYRK kinase discovered, acts as a negative regulator of growth in the budding yeast *Saccharomyces cerevisiae* (Garrett et al., 1991). Pom1 is well studied in the fission yeast *Schizosaccharomyces pombe*, in which Pom1 provides positional information for both growth and division (Bähler and Pringle, 1998). However, the functional roles of Pom1 kinase in fungal development and virulence remain largely unknown in filamentous fungi. This prompted us to investigate the functional roles of Pom1 in *C. scovillei*.

In this study, we identified *C. scovillei POM1* as a homolog of *S. pombe POM1*, which was designated *CsPOM1*. This study aimed to functionally characterize the roles of *CsPOM1* in fungal development and pathogenicity. We generated a targeted deletion mutant of *CsPOM1* and performed phenotypic analysis. The *CsPOM1* deletion mutant exhibited defects in stress tolerance, hydrophobicity, cell division, conidiation, appressorium formation, and virulence. In addition, we found that the expression of glycogen debranching and class II hydrophobin genes was significantly altered in the *CsPOM1* deletion mutant, which might affect the phenotypic defects of the mutant.

## Materials and Methods

### Fungal Strains and Culture Conditions

*Colletotrichum scovillei* strain KC05 (Han et al., 2016) was used as the wild-type (WT) strain in this study. Transformants were selected on TB3 agar (200 g of sucrose, 3 g of yeast extract, 3 g of casamino acids, 10 g of glucose, and 8 g of agar/L) containing 200 µg/ml of hygromycin B (EMD Millipore, USA) or 400 µg/ml of G418 geneticin (Gibco, USA). Complete agar (CMA, 10 g of sucrose, 6 g of yeast extract, 6 g of casamino acids, and 15 g of agar/L) was used for vegetative growth. V8 agar (V8A, 80 ml of V8 juice, 310 µl 10 N NaOH, and 15 g of agar/L) and oatmeal agar (OMA, 50 g oatmeal and 25 g agar/L) were used for conidiation. Experiments were conducted in triplicates and repeated three times. All data were processed with the SigmaStar statistical software package (SPSS Science, Chicago, IL).

### Bioinformatics Analysis

All sequence information used in this study was obtained from the online database CFGP (http://cfgp.snu.ac.kr). Protein sequences of putative glycogen debranching enzyme (CAP_006079), class II hydrophobin (CAP_004537), and dual-specificity tyrosine phosphorylation-regulated kinase Pom1 (CAP_000731) in *C. scovillei* were obtained through BLAST searches conducted using the protein sequences of Gdb1 (AJW05075) in *Saccharomyces cerevisiae*, Mhp1 (MG01173) in *Magnaporthe oryzae*, and Pom1 (NP_592974) in *Schizosaccharomyces pombe*, respectively. Protein sequence alignment of CsPOM1 with related proteins from fungi was performed using the ClustalW program in MEGA 6.0 (Thompson et al., 1994; Tamura et al., 2013). Phylogenetic analysis of obtained protein sequences was performed using a neighbor-joining method (10,000 bootstrap replicates) (Tamura et al., 2013). Domain structure analysis was performed using InterPro Scan v83.0 (http://www.ebi.ac.uk/interpro/) (Mulder et al., 2005). Oligonucleotide primers used in this study were designed using PrimerQuest^®^ Design Tool (http://sg.idtdna.com/site) and synthesized by Bioneer (Daejeon, South Korea).

### Vector Construction for Fluorescence Microscopy

Plasmid pIGPAPA containing a *GFP* gene under the control of the *Neurospora crassa* lyase gene promoter and a geneticin resistance gene (*gen*) cassette under the control of the *A. nidulans trpC* promoter was provided from the Fungal Plant Pathology Laboratory of Seoul National University, Seoul, South Korea (Horwitz et al., 1999; Lee et al., 2008). To generate a CsPOM1-GFP vector, a 5.8-kb PCR product including 1.7 kb of the 5’-flanking promoter region and *CsPOM1* gene sequence was amplified from WT genomic DNA using the primers GFP_Pom1_F/GFP_Pom1_R (**Supplementary Table S1**). The pIGPAPA vector containing a *GFP* gene was linearized by PCR amplification using the primers GFP_Pom1_VF/GFP_Pom1_VR. The amplified 5.8-kb PCR product was cloned into the linearized pIGPAPA vector using an overlap DNA Cloning Kit (Elpis Biotech, Daejeon, South Korea).

### Generation of Knockout Mutant and Gene Expression Analysis

Oligonucleotide primers used for PCR reactions are listed in **Supplementary Table S1**. Genomic DNA was extracted according to a standard method (Sambrook et al., 1989). Approximately 1.5 kb fragments of upstream and downstream of *CsPOM1* were amplified from WT genomic DNA using primers 5F/5R and 3F/3R, respectively. The amplified upstream and downstream fragments of *CsPOM1* were fused to the 1.5-kb *hyg* cassette amplified from plasmid pBCATPH *via* the double-joint PCR method (Yu et al., 2004; Choi et al., 2009). The resultant fusion was finally amplified using primers NF/NR and used as a knockout construct. Protoplast generation and transformation of *C. scovillei* were carried out according to previous work (Shin et al., 2019). Targeted gene deletion was confirmed with Southern blot hybridization and RT-PCR. For complementation, *CsPOM1* fragments including 1.9 kb upstream and 600 bp downstream were amplified from WT genomic DNA using primers CsPom1_cmF/R. The amplified fragments were transformed into protoplasts of *CsPOM1* deletion mutant with plasmid pII99 containing a geneticin resistance gene (*gen*) cassette (Yi et al., 2009).

For quantitative PCR (qPCR), total RNA was extracted using the Easy-Spin Total RNA Extraction Kit (Intron Biotechnology, Seoul, South Korea) from frozen fungal tissues according to the manufacturer’s instructions. First-strand cDNA was synthesized with 5 µg of total RNA using the oligo (dT) primer with the SuperScriptTM III First-Strand Synthesis System kit (Invitrogen™ Life Technologies, CA, USA). qPCR was performed on the StepOne Real-Time PCR System (Applied Biosystems, CA, USA) using the HIPI Real-Time PCR 2x Master Mix (Elpis, Daejeon, South Korea) as previously described (Han et al., 2015). Ct values were normalized to that of *β-tubulin* (CAP_007327) and expressed as relative values, with 1 corresponding to WT (Kim et al., 2009). Experiments were conducted in triplicate and repeated three times.

### Microscopy Methods

*C. scovillei* photographs were routinely obtained using a Carl Zeiss Axio Image A2 microscope (Carl Zeiss Microscope Division, Oberkochen, Germany) equipped with differential interference contrast (DIC) and fluorescence optics. Iodine solution (60 mg of Kl and 10 mg of l_2_/ml of sterile distilled water) was used to stain the glycogen in conidia (Thines et al., 2000). To stain nuclei, diamidino-2-phenylindole (DAPI) (Sigma-Aldrich, USA) was dissolved in sterile distilled water to yield a 10-µg/ml staining solution (10×) (Miyakawa et al., 1984). Conidial septa were stained with a combination of Calcofluor White (1 mg of Calcofluor White M2R and 0.5 mg of Evans blue) (Sigma-Aldrich, USA) and 10% potassium hydroxide (KOH) in a 1:1 ratio.

### Phenotype Analysis

Vegetative growth was evaluated by measuring the diameter of colonies at 6 days postinoculation on CM agar. To assess the role of *CsPOM1* in hydrophobicity of aerial hyphae, 10 µl drops of water or 0.1% sodium dodecyl sulfate (SDS), respectively, were placed on the surface of the *CsPOM1* deletion mutant grown on V8 agar for 5 days in the dark. Conidiation was measured by counting the number of conidia harvested with 5 ml of sterile distilled water from 6-day-old V8 agar under continuous light, using a hemocytometer. Conidial morphology was observed under a microscope, and the conidial length was measured using the ZEN imaging software. Conidial germination and appressorium formation were measured after 6 and 16 h postinoculation of conidial drops (5 × 10^4^ conidia/ml) on coverslips, respectively. To observe appressorium formation from hyphal tips, mycelial agar plugs were obtained from 3-day-old OMA plates and placed on slide glasses. The mycelial agar plugs were covered with coverslips and incubated for 3 days in a moistened box.

### Infection Assays

For appressorial penetration and invasive growth assays, conidial drops (5 × 10^4^ conidia/ml) collected from 5-day-old OMA were inoculated onto the surface of green pepper fruits and incubated in a moistened box. To visualize invasive hyphae, we used GFP-expressing strains generated with the pIGPAPA vector. The fruit surface was peeled off using a razor blade as thin as possible, and the appressorial penetration and invasive growth were examined under a microscope. For the disease development assay, conidial drops (15 × 10^4^ conidia/ml) collected from 5-day-old OMA were inoculated onto the surface of pepper fruits and incubated for 14 days in a moistened box. All experiments were repeated three times with three replicates.

## Results

### Identification of *CsPOM1* in *C. scovillei* KC05

We identified *CsPOM1* (locus CAP_000731), a homolog of *Schizosaccharomyces pombe POM1*, through BLAST searches conducted using the Comparative Fungal Genomics Platform (Bähler and Pringle, 1998; Park et al., 2006). The *CsPOM1* gene was predicted to encode a protein of 1,345 amino acids with a putative protein kinase catalytic domain near the C-terminus, and the protein shared 67.4% (positives 80.9%) amino acid identity with the *S. pombe* Pom1 in their kinase domains. Pom1 is a DYRK subfamily member (Aranda et al., 2011). Several members of the DYRK subfamily, including DYRK1A, DYRK1B (Mirk), DYRK2, DYRK3 (REDK), DYRK4, Yak1, and Pom1, have been identified in eukaryotes (Garrett et al., 1991; Bähler and Pringle, 1998; Goyard et al., 2008; Aranda et al., 2011). Among them, Pom1 and Yak1 were found in fungi (Garrett et al., 1991; Bähler and Pringle, 1998; Goyard et al., 2008). Phylogenetic analysis of CsPOM1 and related proteins in other fungal species revealed that Pom1 homologs form a separate lineage distinct from Yak1 homologs (**Supplementary Figure S1A**). Pom1 homologs of *C. higginsianum*, *Fusarium oxysporum*, *M. oryzae*, *N. crassa*, *Botrytis cinerea*, *A. nidulans*, and *S. pombe* showed 98.0%, 93.3%, 97.7%, 95.3%, 90.6%, and 84.9% amino acid identity, respectively, to CsPOM1 in their kinase domains (**Supplementary Figure S1B**). Domain structure analysis confirmed that all DYRKs contain a DYRK homology (DH) box that regulates kinase activity (Becker and Joost, 1998) in the N-terminal of the catalytic domain (**Supplementary Figure S1B**).

### Generation of the *ΔCspom1* mutant

We generated targeted gene deletion mutant *ΔCspom1* to characterize the functions of *CsPOM1* in fungal development and pathogenicity (**Supplementary Figure S2A**). Approximately 120 putative deletion transformants for the *CsPOM1* gene were selected on TB3 agar containing hygromycin B (200 ppm). Among the putative transformants, five candidates were selected by screening PCR, and the *ΔCspom1* mutant was finally selected based on Southern blot hybridization (**Supplementary Figure S2B**). RT-PCR confirmed that the expression of the *CsPOM1* gene was completely abolished in the deletion mutant (**Supplementary Figure S2C**). The expression of the *CsPOM1* gene was restored in the complemented strain.

### Vegetative Growth of the *ΔCspom1* Mutant

**Figure 1:**
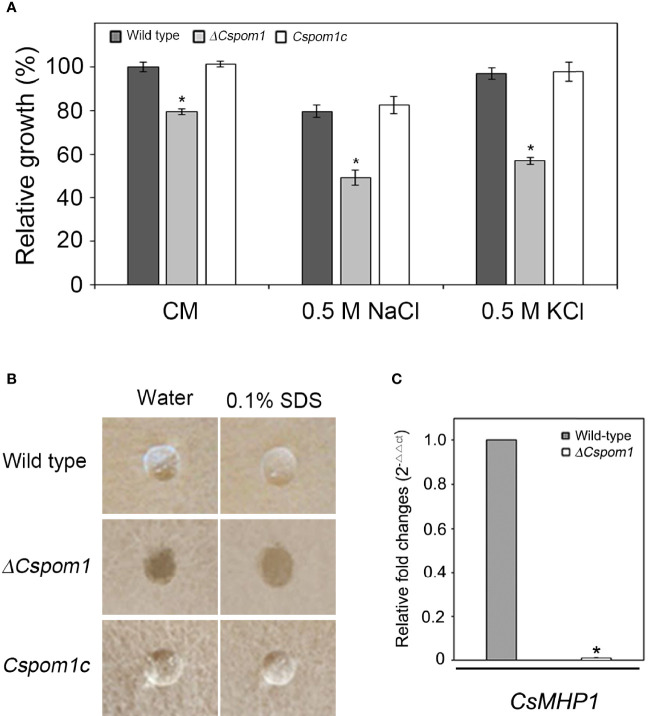
Vegetative growth and surface hydrophobicity of the *ΔCspom1* mutant. **(A)** Fungal colonies were grown on CM agar with and without an osmotic stress agent (0.5 M NaCl or KCl) for 6 days at 25°C in the dark. Relative growth was compared with WT grown on CM agar. Asterisk indicates significant difference between WT and mutant according to Tukey’s test at *p* < 0.05. **(B)** First, 10 µl water or 0.1% SDS were placed on the surface of each strain grown on V8 agar for 5 days in the dark. **(C)** Measurement of the expression of the *CsMHP1* gene encoding a putative class II hydrophobin (CAP_004537) in the *ΔCspom1* mutant. Expression of the *CsMHP1* gene was measured by qPCR, normalized to *β-tubulin*, and expressed as a relative value of 1 in the WT.

To investigate the roles of *CsPOM1* in the vegetative growth of *C. scovillei*, we measured the vegetative growth of the wild-type (WT) and *ΔCspom1* mutant on CM agar. After 6 days of incubation, the *ΔCspom1* mutant exhibited reduced vegetative growth compared with WT (20% reduction in average) (**Figure 1A**). Tolerance to environmental stress is important for the normal growth of fungi (Huang et al., 2017). To investigate whether *CsPOM1* is involved in stress tolerance, we inoculated mycelial agar plugs of the WT and mutant on CM agar supplemented with an osmotic stress agent, followed by incubation for 6 days. Relative growth was compared with WT on CM agar without supplements. The vegetative growth of mutant on CM agar supplemented with 0.5 M NaCl or 0.5 M KCl (osmotic stress) was significantly reduced compared with WT (38% and 41% reduction, respectively), which exhibits more severe reductions than that on CM agar without supplements (20% reduction) (**Figure 1A**). The results indicate that *CsPOM1* is involved in the tolerance to osmotic stress of *C. scovillei*.

### Surface Hydrophobicity of the *ΔCspom1* Mutant

To assess the role of *CsPOM1* in the surface hydrophobicity of *C. scovillei*, 10 µl drops of water or 0.1% SDS, respectively, were placed on the surface of WT and *ΔCspom1* mutant grown on V8 agar for 5 days. We found that the drops of water and 0.1% SDS were suspended on the aerial hyphae of the WT, whereas they were immediately soaked into the mycelia of *ΔCspom1* (**Figure 1B**). We identified a putative class II hydrophobin gene *CsMHP1* (CAP_004537) in *C. scovillei*, a homolog of *M. oryzae MHP1* (MG01173) sharing 46.1% amino acid identity (Kim et al., 2005), and compared the relative gene expression by quantitative PCR (qPCR). We found that expression of the *CsMHP1* gene was significantly reduced in the *ΔCspom1* mutant compared to WT (**Figure 1C**). Collectively, these results indicate that *CsPOM1* is required for surface hydrophobicity, which is likely associated with the expression of the *CsMHP1* gene.

### *CsPOM1* Is Important for Conidial Production, Conidial Morphology, and Glycogen Metabolism

**Figure 2:**
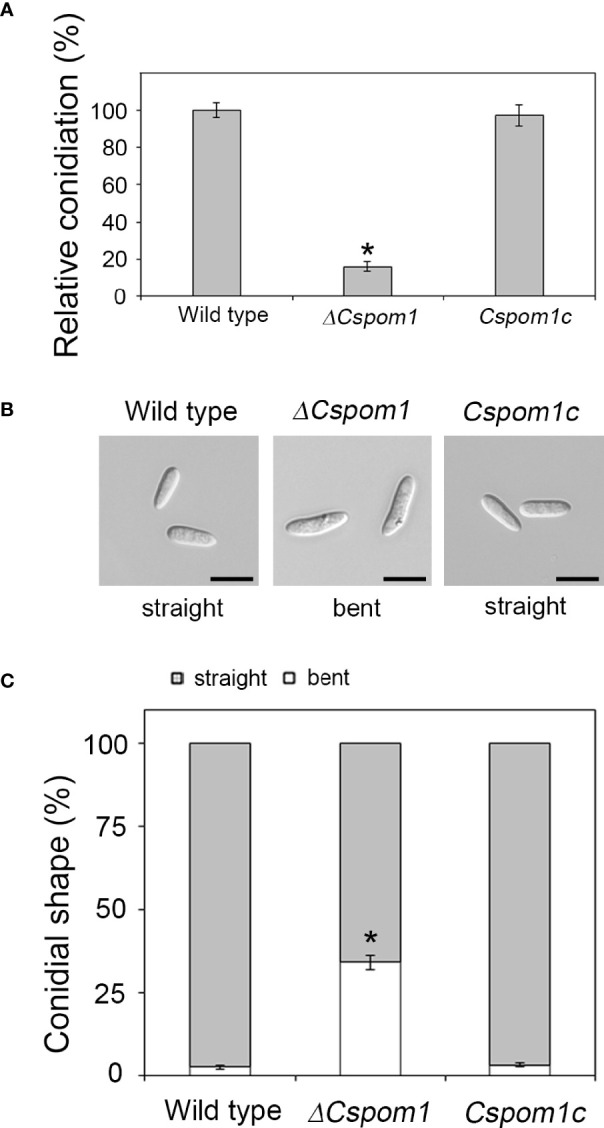
Abnormal conidial production of the *ΔCspom1* mutant. **(A)** Quantitative measurement of conidia at 6 days postinoculation on V8 agar at 25°C under continuous light. Asterisk indicates a significant difference at *p* < 0.05 according to Tukey’s test. **(B)** Conidial morphology of the ***Δ*** *Cspom1* mutant. Scale bars, 10 µm. **(C)** Quantitative comparison of abnormal conidia (bent shape). Conidia were harvested from 10-day-old OMA. The shape of the conidia was observed under a microscope. Asterisk indicates a significant difference at *p* < 0.05 according to Tukey’s test.

Conidial production of the *ΔCspom1* mutant was quantified on V8 agar. Quantitative analysis of conidial production showed that the *ΔCspom1* mutant produced significantly fewer conidia than WT (**Figure 2A**). This phenotype of the *ΔCspom1* mutant was rescued in complemented strain *Cspom1c*. Next, we collected conidia of the mutants from 10-day-old OMA and examined the conidial morphology. The *ΔCspom1* mutant produced longer conidia (15.3 ± 0.17 in length) than those of the WT (10.3 ± 0.16 μm in length) (**Figure 2B**). In addition, approximately 34% of the *ΔCspom1* mutant conidia were slightly bent, while most of the WT conidia were straight in shape (**Figures 2B, C****)**. Collectively, these data indicate that *CsPOM1* is involved in the regulation of conidial production and conidial size.

**Figure 3:**
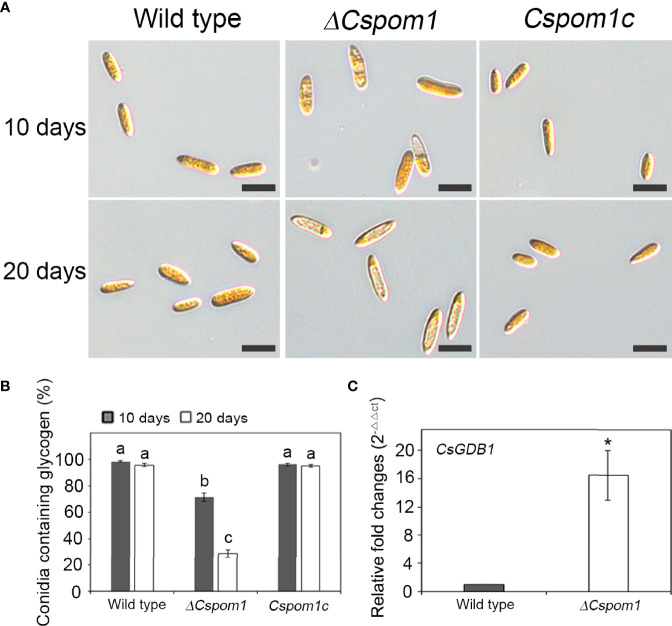
Glycogen metabolism of the *ΔCspom1* mutant in conidia. **(A)** Conidia harvested from 10- or 20-day-old OMA were stained with iodine solution (2×, 60 mg of Kl and 10 mg of l_2_/ml of sterile distilled water). Scale bars, 10 µm. **(B)** Quantitative measurement of conidia containing glycogen. Different letters on bars indicate significant differences at *p* < 0.05 according to Tukey’s test. **(C)** Measurement of the expression of the *CsGDB1* gene encoding a putative glycogen debranching enzyme (CAP_006079) in the *ΔCspom1* mutant. Expression of the *CsGDB1* gene was measured by qPCR, normalized to *β-tubulin*, and expressed as a relative value of 1 in the WT. Asterisk indicates a significant difference between the WT and mutant at *p* < 0.05 according to two-tailed *t*-test.

We also examined glycogen deposits in conidia. Iodine staining of 10-day-old conidia revealed that only 71% of the *ΔCspom1* mutant conidia contained glycogen, compared with more than 90% of WT conidia (**Figures 3A, B****)**. Longer incubation (up to 20 days) further decreased the rate of glycogen-containing conidia, down to 29% in the *ΔCspom1* mutant, while most of the WT conidia still contained glycogen (**Figures 3A, B****)**. We identified a putative glycogen debranching gene in *C. scovillei*, i.e., *CsGDB1*, which is a homolog of *S. cerevisiae GDB1* (AJW05075) sharing 47.0% amino acid identity, and compared the relative gene expression during vegetative growth in CM broth by qPCR. The qPCR analysis confirmed that expression of the glycogen debranching gene (CAP_006079) was significantly higher in the *ΔCspom1* mutant compared with WT (**Figure 3C**). Taken together, these results indicate that *CsPOM1* is involved in the regulation of glycogen metabolism in a conidial age-dependent manner.

### *CsPOM1* Regulates Conidial Cell Division and Is Localized in the Region of Septum Formation

**Figure 4:**
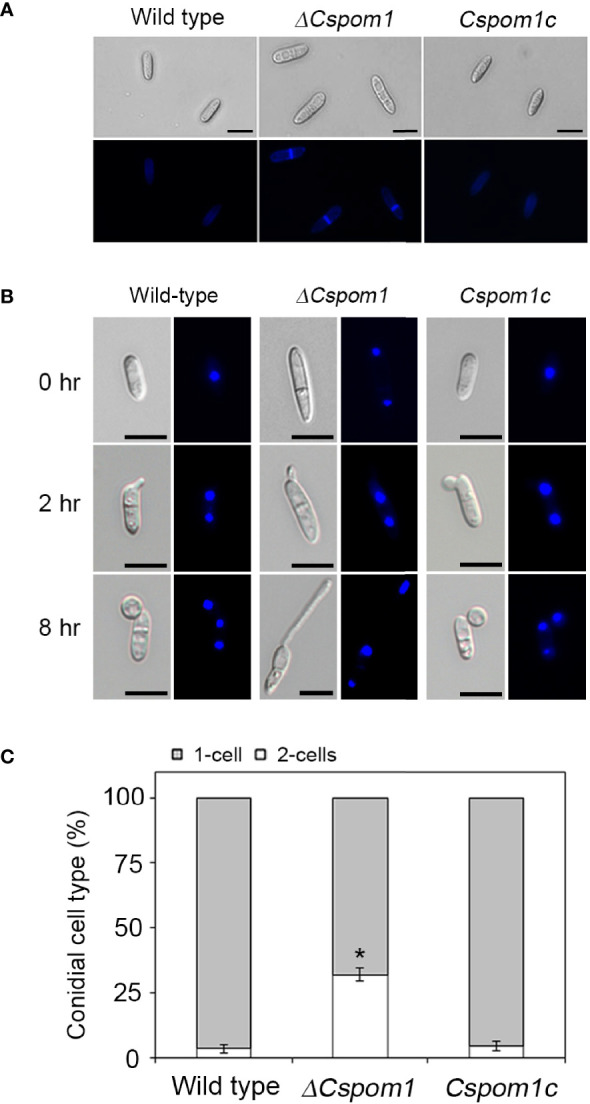
Conidial cell division of the *ΔCspom1* mutant. **(A)** Septation of conidia on coverslips (0 h). The conidial septa were stained with Calcofluor White (1 mg Calcofluor White M2R and 0.5 mg Evans blue). Scale bars, 10 µm. **(B)** Nuclear division during conidial germination and appressorium development on coverslips. The conidial nuclei were stained with DAPI (1 µg/ml). Scale bars, 10 µm. **(C)** Quantitative comparison of the number of conidial cells (one- or two-celled) between the WT and mutant. Experiments were conducted in triplicate and repeated three times (*n* ≥ 100 conidia per strain). Asterisk indicates a significant difference according to Tukey’s test at *p* < 0.05.

Pom1 kinase regulates cell division in the fission yeast *S. pombe* (Bähler and Pringle, 1998). Therefore, we examined conidial septation and nuclear division during appressorium formation on hydrophobic coverslips. The WT conidia were aseptate and uninucleate at 0 h, which was visualized by Calcofluor White (**Figure 4A**) and DAPI staining (**Figure 4B**), respectively. By 2 h, the conidia formed two uninuclear cells separated by a septum, and a single germ tube emerged (**Figure 4B**). The tip of the germ tube began to swell and produced an appressorium. A second round of nuclear division started, resulting in another nucleus in the appressorium by 8 h (**Figure 4B**). However, approximately 32% of the *ΔCspom1* conidia were already septate (**Figure 4A**) and two-celled (**Figure 4B**) at 0 h, in which most of the two-celled conidia were slightly bent in shape. These results suggest that *CsPOM1* may regulate conidial cell division in *C. scovillei*.

**Figure 5:**
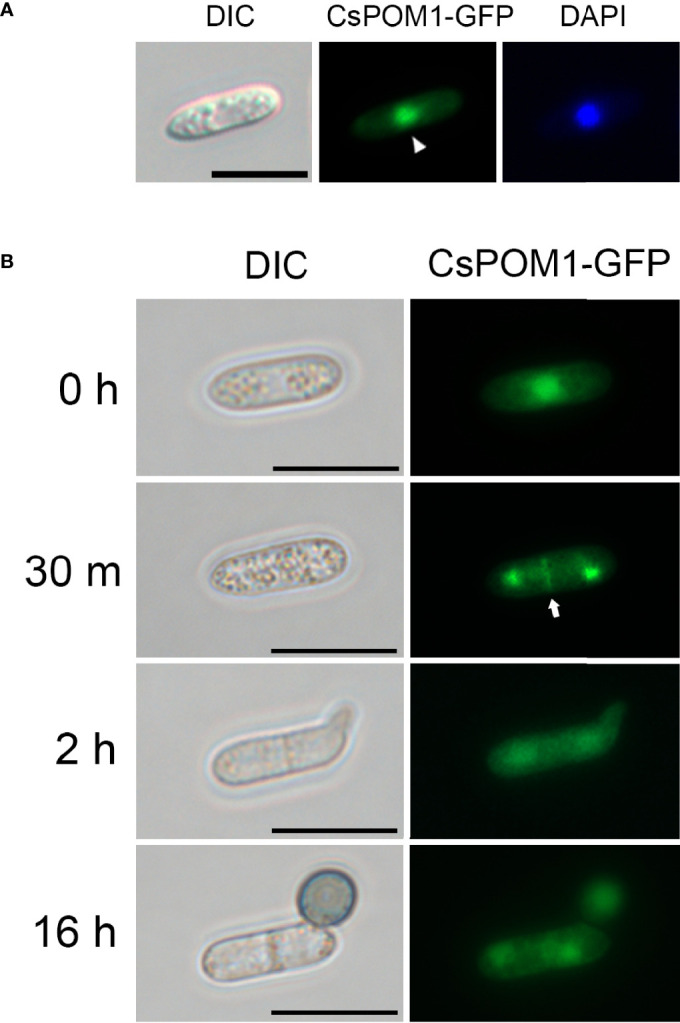
Intracellular localization of CsPom1-GFP fusion. Wild-type conidia expressing CsPom1-GFP fusion were inoculated on coverslips and incubated in a moistened box. **(A)** Nuclear localization of CsPOM1-GFP fusion. The conidial nuclei were stained with DAPI (1 µg/ml). Scale bar,10 µm. **(B)** Localization of CsPOM1-GFP fusion during conidial germination and appressorium formation. Arrow indicates the region of septum formation in conidia. Scale bars, 10 µm.

Next, we tagged the 3’ end of *CsPOM1* with sequences encoding a green fluorescent protein (GFP) and transformed the construct into WT protoplasts to investigate the intracellular localization of CsPOM1. Conidia of the WT transformant were inoculated on hydrophobic coverslips, and the GFP-tagged Pom1 was monitored. The fluorescent analysis demonstrated that CsPom1-GFP was localized to the nucleus in conidia (**Figure 5A**). The GFP signal at the nucleus in conidia became weaker during germination (**Figure 5B**). CsPOM1-GFP was also observed in the region of septum formation in conidia at 30 min postinoculation (**Figure 5B**). The GFP signal was very faint and became weaker during germination (**Figure 5B**).

### *CsPOM1* Is Essential for Appressorium Formation in Conidia and Hyphal Tips

**Figure 6:**
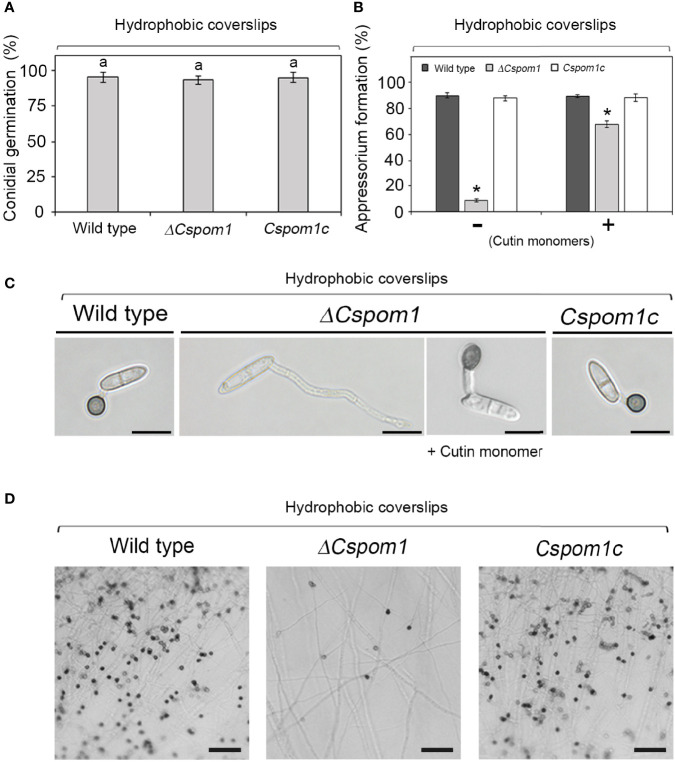
Appressorium formation of the *ΔCspom1* mutant. **(A)** Quantitative measurement of conidial germination at 6 h postinoculation on coverslips. Experiments were conducted in triplicate and repeated three times (*n* ≥ 100 conidia per strain). The same letters on bars indicate there was no significant difference according to Tukey’s test. **(B)** Quantitative measurement of appressorium formation of conidia with or without 10 μM cutin monomers at 16 h postinoculation on coverslips. Experiments were conducted in triplicate and repeated three times (*n* ≥ 100 conidia per strain). Asterisks indicate significant differences according to Tukey’s test at *p* < 0.05. **(C)** Appressorium formation of conidia on coverslips. Scale bars, 10 µm. **(D)** Appressorium formation from hyphal tips. Mycelial agar plugs from 3-day-old OMA were placed on slide glasses and covered with coverslips. Photographs were taken at 3 days postinoculation on coverslips. Scale bars, 50 µm.

To investigate the functional role of *CsPOM1* in appressorium formation, we collected conidia of the WT and mutants (5 × 10^5^ conidia/ml) from 6-day-old OMA plates and inoculated conidial drops on coverslips. The experiment revealed that the *ΔCspom1* mutant was normal in germination but defective in appressorium formation on hydrophobic coverslips (**Figures 6A, B****)**. At 16 h postinoculation, approximately 90% of the WT conidia formed melanized appressoria (**Figures 6B, C****)**. However, only 8% of the *ΔCspom1* mutant conidia formed appressoria (**Figures 6B, C****)**. Similarly, appressorium formation from hyphal tips was significantly reduced in the *ΔCspom1* mutant (**Figure 6D**). These results suggest that *CsPOM1* is essential for appressorium formation from germ tube and hyphal tips. Treatment of conidia with synthetic cutin monomers or exogenous cyclic adenosine monophosphate (cAMP) is a method to induce appressorium formation in some fungi (Lee and Dean, 1994; Fu et al., 2021). We treated *ΔCspom1* mutant conidia with 10 μM cutin monomers and 5 mM exogenous cAMP, respectively. The cutin monomer treatment partially restored appressorium formation of the *ΔCspom1* mutant (**Figures 6B, C****)**. In contrast, the cAMP treatment did not restore appressorium formation of the *ΔCspom1* mutant (**Supplementary Figure S3**), which indicates that CsPOM1 functions downstream of cAMP or in a cAMP-independent manner.

### *CsPOM1* Is Required for Early Infection

**Figure 7:**
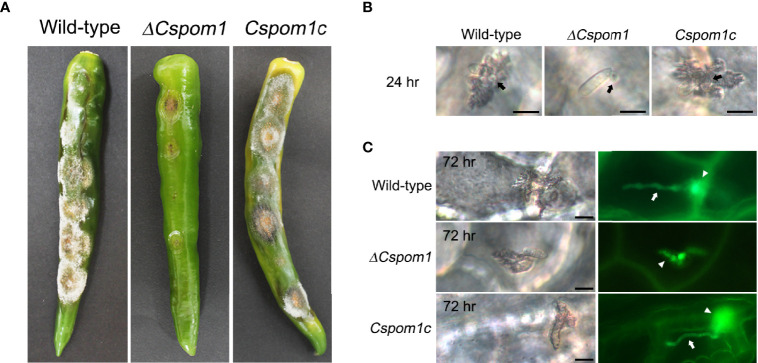
CsPom1 is important for plant penetration and invasive growth. **(A)** Conidial drops (15 × 10^4^ conidia/ml) of WT and *ΔCspom1* mutant were inoculated onto the surface of green pepper fruits (*Capsicum annuum* L. cv. Gilsang). Photographs were taken at 14 days postinoculation. **(B)** Green pepper fruits were inoculated with conidial drops (5 × 10^4^ conidia/ml) of the WT and ***Δ*** *Cspom1* mutant. Photographs were taken at 24 h postinoculation **(B)** and 72 h postinoculation **(C)**. Black arrows indicate appressorium. White arrows indicate primary hypha in a plant epidermal cell. White arrowheads indicate fungal hypha in plant cuticle. Scale bars, 10 µm.

Next, we investigated the roles of *CsPOM1* in fungal virulence on pepper fruit. We inoculated conidial drops (15 × 10^4^ conidia/ml) collected from 5-day-old OMA plates onto the surface of pepper fruits (*C. annuum* L. cv. Gilsang) and incubated them in a moistened box. After 2 weeks, the WT caused typical anthracnose lesions on the pepper fruits, while the *ΔCspom1* mutant produced tiny lesions on the fruits (**Figure 7A**). To investigate initial pepper fruit infection by the mutant, we generated GFP-expressing transformants and inoculated conidia on pepper fruits. Most of the WT conidia (>90%) formed appressoria on the pepper fruits, penetrated the plant cuticle by 24 h (**Figure 7B**), and formed a primary hypha in the plant epidermal cell by 72 h (**Figure 7C**). Unlike the appressorium formation on coverslips (**Figure 6B**), most of the *ΔCspom1* conidia (>80%) formed appressoria on the pepper fruits by 24 h, although there was a slight reduction in appressorium formation compared with the WT (**Supplementary Figure S4**). However, the mutant conidia exhibited delayed penetration and invasive growth during infection compared with WT (**Figures 7B, C****)**. Collectively, these results suggest that *CsPOM1* is involved in penetration and invasive growth during early infection on pepper fruits.

## Discussion

The DYRK kinase Pom1 is well-known to provide positional information for cell division in the fission yeast *S. pombe*, but the roles of Pom1 in filamentous fungi are not well characterized. In this study, we functionally characterized *CsPOM1*, a homolog of *S. pombe POM1*, in the anthracnose fungus *C. scovillei*. The *ΔCspom1* mutant exhibited reduced vegetative growth on CM agar compared with the WT (**Figure 1A**). In the fission yeast *S. pombe*, deletion of *POM1* results in failure of switching from unipolar to bipolar growth (Bähler and Pringle, 1998). In the filamentous fungus *F. graminearum*, the vegetative growth of the *Fgpom1* mutant was slightly reduced on potato dextrose agar media (Wang et al., 2011). Thus, our results agree with previous reports that *POM1* is involved in cell growth. In addition, the growth defect of the *ΔCspom1* mutant became more severe when it was exposed to osmotic stress (NaCl or KCl). This suggests that *CsPOM1* is involved in osmoregulation, which might affect the basic growth phenotype of the *ΔCspom1* mutant.

Hydrophobins are small, hydrophobic proteins that provide a hydrophobic coating on aerial hyphae and conidia in fungi (Wessels et al., 1991; Kim et al., 2005; Pham et al., 2016). The hydrophobic surfaces of fungal pathogens are associated with conidial production, and the conidia surfaces are coated to reduce wettability and promote surface interaction (Pham et al., 2016). In *M. oryzae*, deletion of *MHP1*, a class II hydrophobin gene, results in a loss of surface hydrophobicity and reduction in conidiation (Kim et al., 2005). In this study, the *ΔCspom1* mutant exhibited a loss of hydrophobicity, reduced conidiation, and reduced expression of *CsMHP1* compared with WT (**Figures 1**, **2**). Thus, we suggest that *CsPOM1* is involved in the transcriptional regulation of the *CsMHP1* gene in *C. scovillei*, in which the reduced expression of the *CsMHP1* gene caused the defects in surface hydrophobicity and conidiation in the *ΔCspom1* mutant.

Conidia of *C. scovillei* were uninucleated single cells that became two-celled just before germination and differentiated into an appressorium with one nucleus (**Figures 4A, B****)**. In the fission yeast *S. pombe*, Pom1 forms a polar gradient at the cell ends inhibiting Cdr2 activity, which causes a delay in mitotic entry (Moseley et al., 2009; Gerganova et al., 2019). Deletion of *POM1* in *S. pombe* results in premature entry into mitosis (Moseley et al., 2009). Consistently, approximately 32% of the conidia produced by the *ΔCspom1* mutant were already two-celled before inoculation of the conidia on an inductive surface (**Figure 4A–C**). Thus, we suggest that *CsPOM1* has an important role in the negative regulation of cell division in *C. scovillei*. In *S. pombe*, Pom1 is localized at both cell ends and also localized to the cell division site (Bähler and Pringle, 1998). As cell division proceeds, the concentration of Pom1 at both cell ends decreases while the concentration at the cell center increases (Bähler and Pringle, 1998; Celton-Morizur et al., 2006). In our study, we found that CsPOM1-GFP fusion is localized in the region of septum formation in conidia after a 30-min incubation on hydrophobic coverslips, which is approximately the time at which nuclear division starts (**Figure 5B**). Consistent with *S. pombe* Pom1, this suggests that CsPOM1 provides positional information for cell division in *C. scovillei*. Unlike the *S. pombe* Pom1, the localization of CsPOM1-GFP fusion in the nuclei of ungerminated conidia were noticeable (**Figure 5A**), although some DYRK family kinases, including DYRK1A and Yak1, are localized to the nuclei (Moriya et al., 2001; Kaczmarski et al., 2014; Han et al., 2015). This may reflect divergent roles for *CsPOM1* in the filamentous fungus, as shown in pleiotropic defects of the *ΔCspom1* while the cellular roles of the protein await elucidation.

Glycogen is one of the major energy stores in fungi (Talbot, 1995). Glycogen is utilized during conidial germination and appressorium development, and is involved in the supply of energy during infection in fungi (Thines et al., 2000; Badaruddin et al., 2013). In our study, glycogen deposits in conidia of the *ΔCspom1* mutant were already degraded before conidial germination (**Figures 3A, B****)**. In addition, the mutant exhibited delayed plant penetration, resulting in tiny and restricted lesions on pepper fruits (**Figure 7**). We found that expression of the *CsGDB1* gene in the *ΔCspom1* mutant was significantly increased compared with WT (**Figure 3C**). Thus, it is likely that *CsPOM1* acts as a negative regulator inhibiting the expression of the glycogen debranching gene, *CsGDB1* in *C. scovillei*, which is likely associated with the abnormal glycogen degradation and reduced virulence in the *ΔCspom1* mutant.

Many phytopathogenic fungi recognize various host signaling factors including surface hydrophobicity, cutin monomers, and leaf waxes to develop appressoria (Adachi and Hamer, 1998; Kang et al., 1999; Dickman et al., 2003). In this study, the *ΔCspom1* mutant exhibited significantly reduced appressorium formation on hydrophobic coverslips, but the defect was restored on the plant surface (**Supplementary Figure S4**) and by cutin monomer treatment (**Figure 6**). Therefore, we think that *CsPOM1* contributes to the recognition of hydrophobic surface for appressorium formation in *C. scovillei*. In rice blast fungus *M. oryzae*, targeted deletion of class I hydrophobin *MPG1* or class II hydrophobin *MHP1* results in reduced appressorium formation on hydrophobic surface, and infectious growth in plant cells is significantly limited (Talbot et al., 1993; Kim et al., 2005). The addition of cAMP restored appressorium formation in the *MPG1* deletion mutant, but not in the *MHP1* deletion mutant, indicating that Mhp1 may function downstream of cAMP for appressorium formation in *M. oryzae* (Talbot et al., 1993; Kim et al., 2005). In our study, the addition of cAMP did not restore appressorium formation in the *ΔCspom1* mutant (**Supplementary Figure S3**). Considering that the *ΔCspom1* mutant exhibited significantly reduced expression of the *CsMHP1* gene, *CsPOM1* may function downstream of cAMP or a cAMP-independent manner for appressorium formation in *C. scovillei*. On pepper fruits, the *ΔCspom1* mutant was able to form appressoria, probably *via* the sensing of signals on the plant surface, but the mutant showed a delay in penetration and infectious growth in plant cells (**Figure 7**). Therefore, the reduction of *ΔCspom1* in virulence may be related to the defects in plant penetration and growth of invasive hyphae after penetration. *POM1* has not been studied in the context of appressorium formation in other fungi, but the *YAK1* kinase gene, belonging to the DYRK subfamily with *POM1*, is required for appressorium formation on the hydrophobic surface in *M. oryzae* (Han et al., 2015). Similar to the *ΔCspom1* mutant, the *ΔMoyak1* mutant in *M. oryzae* was able to form appressoria on the plant surface, but the appressoria were defective in plant penetration. Thus, these two genes belonging to the DYRK subfamily appear to have similar roles in the appressorium formation of phytophathogic fungi.

Based on the pleiotropic roles and nuclear localization of *CsPOM1*, we speculate that *CsPOM1* may play diverse roles in phosphorylating regulators in signaling pathways for fungal development and metabolism. Supportively, a recent study reported that PomA regulates the HOG MAPK pathway in *A. nidulans* (Zhou et al., 2019). Two HOG MAPKs, SakA and MpkC, regulate the expression of *gdbA*, a glycogen debranching gene, during glycogen metabolism in *A. fumigatus* (de Assis et al., 2018). Our study revealed that *CsPOM1*, a DYRK kinase gene, plays important roles in fungal morphogenesis and disease development in *C. scovillei*, which provide fundamental basis to understand anthracnose diseases on fruits caused by *Colletotrichum* species.

## Data Availability Statement

The raw data supporting the conclusions of this article will be made available by the authors, without undue reservation.

## Author Contributions

Conceived and designed the experiments: JHS, HYK, TF, KHL, KSK. Performed the experiments: JHS, HYK, TF, KHL. Analyzed the data: JHS, HYK, TF, KSK. Wrote the paper: JHS, KSK. Originated research leading up to this paper and provided guidance and review: JHS, HYK, KSK.

## Funding

This study was supported by Basic Science Research Program through the National Research Foundation of Korea grant (NRF-2020R1A2C100550700) funded by the Ministry of Education, Science and Technology.

## Conflict of Interest

The authors declare that the research was conducted in the absence of any commercial or financial relationships that could be construed as a potential conflict of interest.

## Publisher’s Note

All claims expressed in this article are solely those of the authors and do not necessarily represent those of their affiliated organizations, or those of the publisher, the editors and the reviewers. Any product that may be evaluated in this article, or claim that may be made by its manufacturer, is not guaranteed or endorsed by the publisher.
