# Supplementary material for: CsPOM1, a DYRK Family Kinase, Plays Diverse Roles in Fungal Development, Virulence, and Stress Tolerance in the Anthracnose Pathogen Colletotrichum scovillei
Source: Front Cell Infect Microbiol. 2022 Apr 26;12:861915. doi: 10.3389/fcimb.2022.861915 (PMC9088010; doi:10.3389/fcimb.2022.861915)
Supplement: Supplementary file 1 [file DataSheet_1.docx]

Supplementary Material


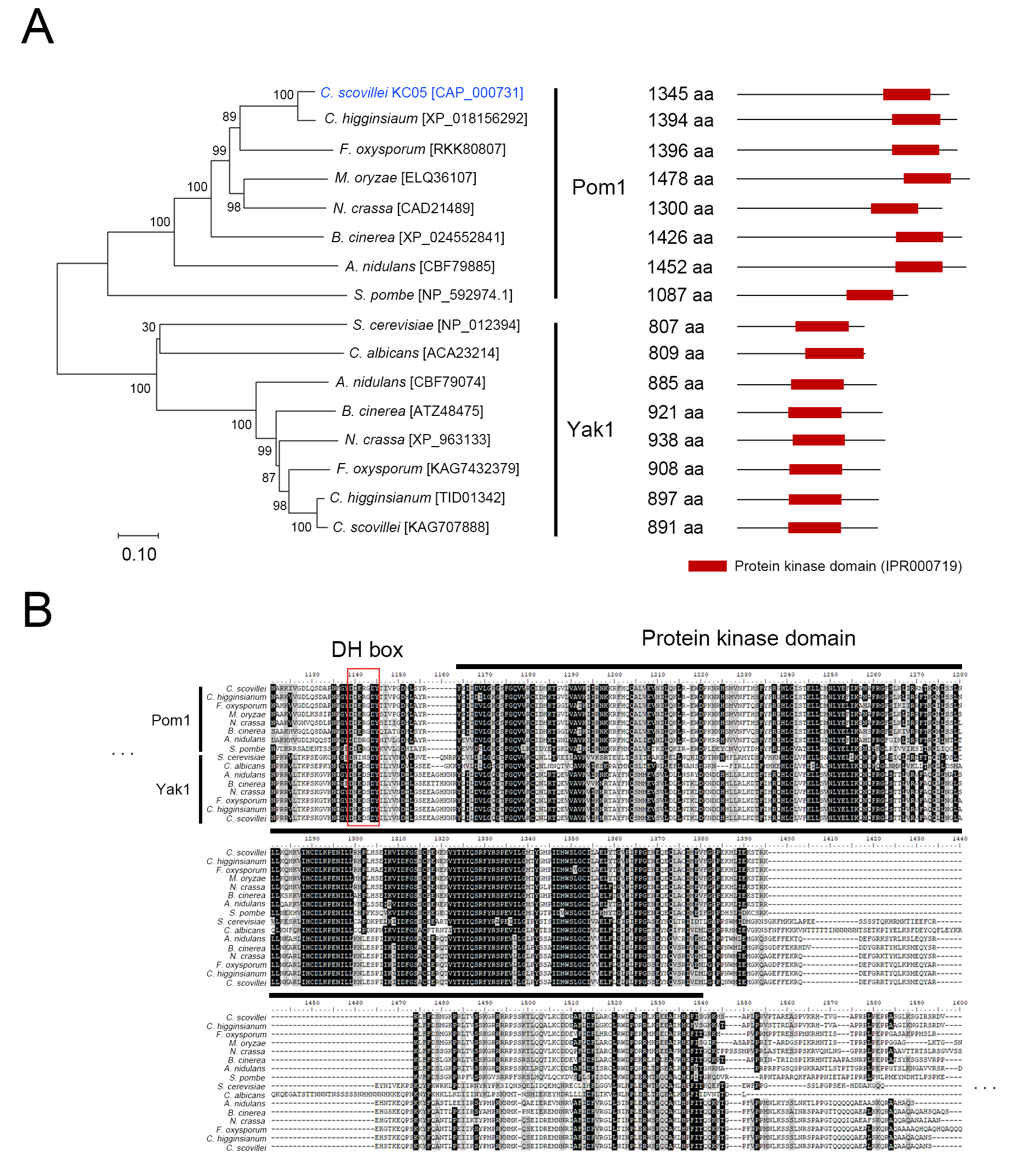


**Fig. S1. Phylogenetic analysis of Pom1 homologs and related DYRK subfamily members.** (A) A neighbor-joining tree was constructed using the MEGA 7 program. The scale bar represents the number of amino acid difference per site. (B) Amino acid sequence alignment of the protein kinase domain was performed using ClustalW in MEGA 6.0. Identical amino acids and conserved substitutions are shaded in black and gray, respectively. The red box represents a DYRK homology (DH) box. Black line represents a protein kinase domain.


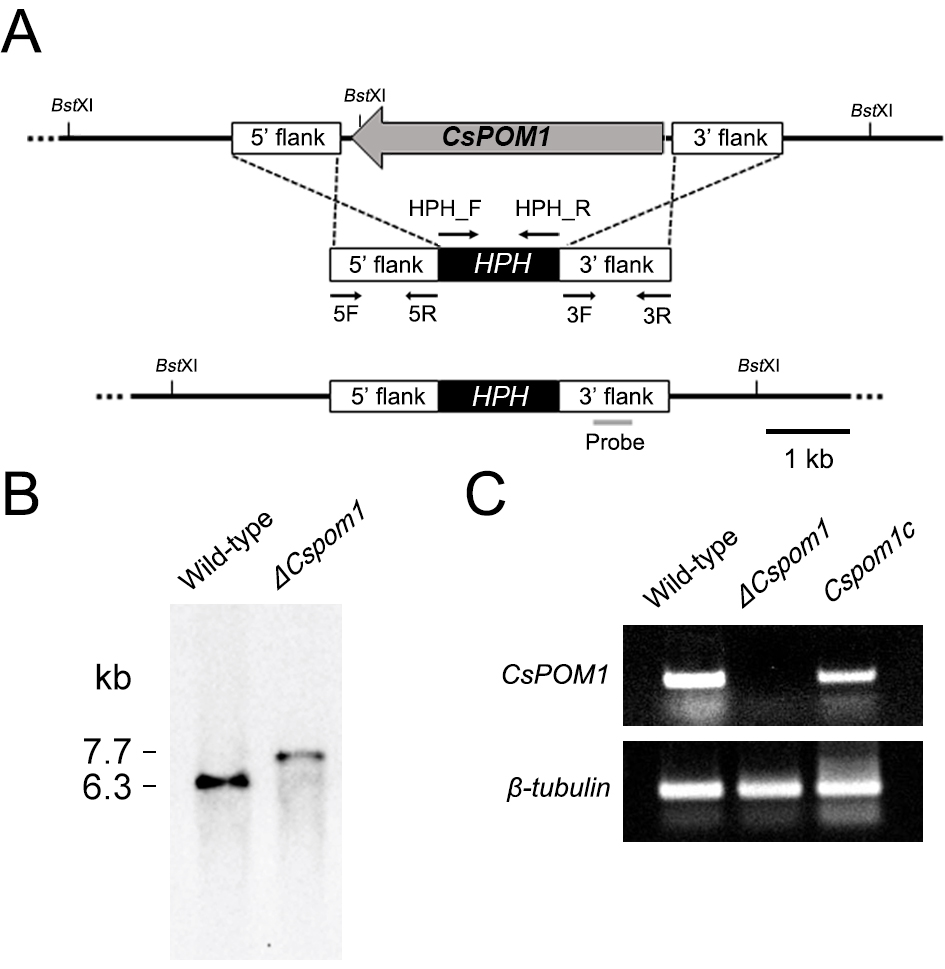


**Fig. S2.** **Targeted deletion of *CsPOM1* gene.** (A) Schematic representation of the targeted gene deletion strategy of *CsPOM1* in *C. scovillei*. Double-joint PCR was performed to generate the gene deletion construct. *Bst*XI was used to digest genomic DNA. Digested genomic DNA was hybridized to a probe. Southern blotting (B) and RT-PCR (C) were performed to confirm deletion of the gene.


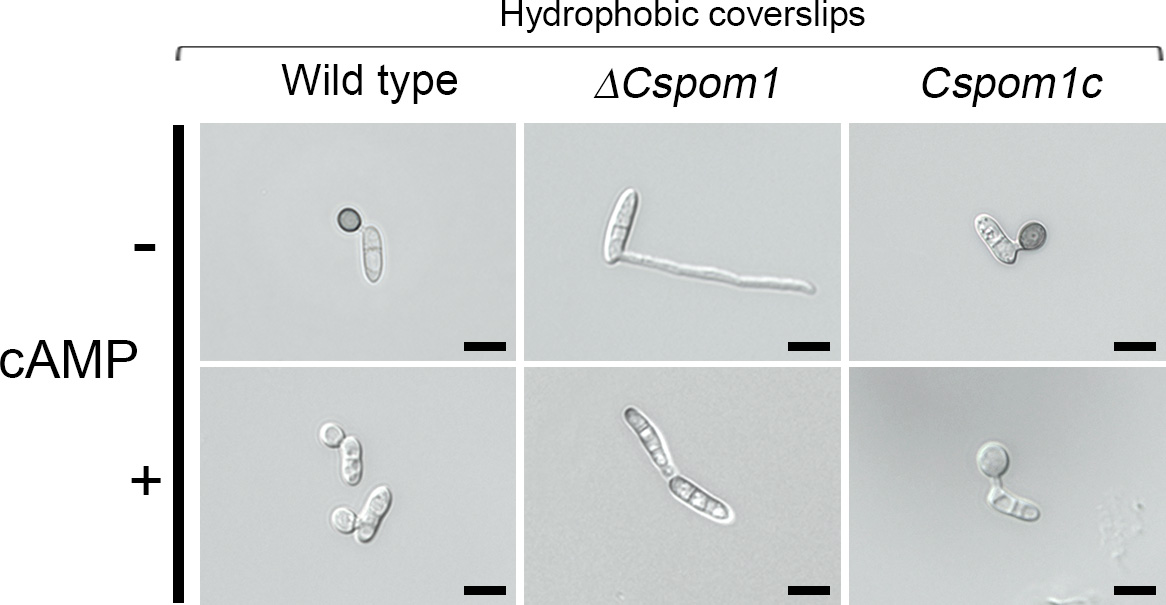


**Fig. S3.** **Appressorium formation of the *ΔCspom1* mutant on artificial surface.** Drops of conidial suspension (5 × 10^4^ conidia/mL) were placed on hydrophobic cover slips and incubated for 16 h; exogenous cAMP (5 mM) was added to the conidial drops at 2 h post-inoculation. Scale bars = 10 µm.


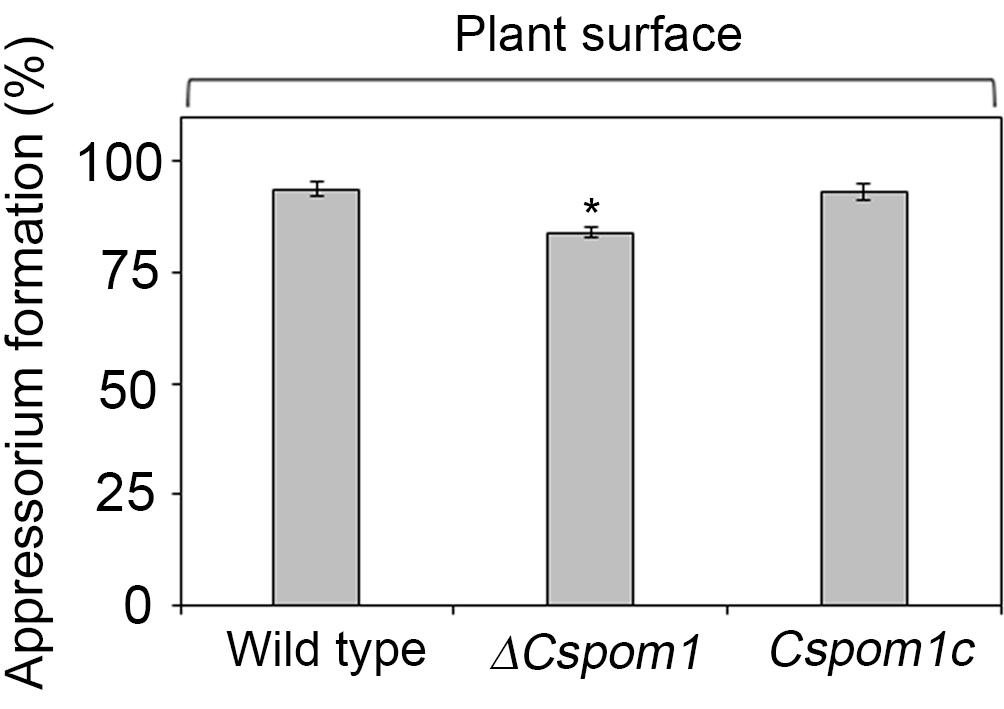


**Fig. S4.** **Appressorium formation rate of the *ΔCspom1* mutant on plant surface.** Drops of conidial suspension (5 × 10^4^ conidia/mL) were placed on the surface of green pepper fruits and incubated for 24 h. Experiments were conducted in triplicate and repeated three times (n ≥ 100 conidia per strain). Asterisks indicate a significant difference according to Tukey’s test at p < 0.05.

**Supplementary Table 1**. Primers used in this study

| Name | Primer sequence 5’to 3’ | | | | |
| --- | --- | --- | --- | --- | --- |
| **CsPOM1** | |  | | | |
| CsPOM1_5F | | AGACAATGAGCGATCCAAGAC | | | |
| CsPOM1_5R | | CCTCCACTAGCTCCAGCCAAGCCCCCTTACGAACAGCGAACAT | | | |
| CsPOM1_3F | | GTTGGTGTCGATGTCAGCTCCGGAGGGTTTGCGAGTCTGGGATT | | | |
| CsPOM1_3R | | GGGAGTTGCGTACCTAATTGA | | | |
| CsPOM1_NF | | CACTCGTCATCAGGTCCATT | | | |
| CsPOM1_NR | | TGCTCTCTCTTTCTCACTCTTG | | | |
| CsPOM1_SF | | ACGTCTTCCGTGTGTGTTTAT | | | |
| CsPOM1_SR | | GTTCACGCCGCTTTGTTATG | | | |
| CsPOM1_PF | | CTCATTCCGGGACAACA | | | |
| CsPOM1_PR | | CAAGAAACAACGAGGAAGAG | | | |
| CsPOM1_RTF | | GGTCCAGTTCTCACTCTT | | | |
| CsPOM1_RTR | | AGCGTGTCTCCATTCTT | | | |
| CsPOM1_cmF | | GGAGCGTCCATTCGTTAGAA | | | |
| CsPOM1_cmR | | GCGCAATTCGTGCAATCA | | | |
| ***β*-tubulin** | |  | | | |
| TUB_F | | AAGCTCGCCGTCAACATGG | | | |
| TUB_R | | CGACGGAACATGGCAGTGAA | | | |
| **Vector construction** | |  | | | |
| GFP_POM1_F | | TCGATTCAGATGGATGTTTCCC | | | |
| GFP_POM1_R | | CAAACCCCTCCGACTCATGGC | | | |
| GFP_POM1_VF | | AGTCGGAGGGGTTTGATGGTGAGCAAGGGCGAGGA | | | |
| GFP_POM1_VR | | ATCCATCTGAATCGACAACATACGAGCCGGAAGCA | | | |
| **Hygromycin B phosphotransferase** | | | |  | |
| HPH_R | | GGCTTGGCTGGAGCTAGTGGAGG | | | |
| HPH_R | | CTCCGGAGCTGACATCGACACCAAC | | | |
| **G418** | |  | | | |
| Gen_F | | AGAAGATGATATTGAAGG | | | |
| Gen_R | | CTCTAAACAAGTGTACCTGTGC | | | |
| **Glucose debranching enzyme** | | | | |  |
| CsGDB1_QF | | CGGCAAGCTCGTTTATG | | | |
| CsGDB2_QR | | GCGTCCGACAATGTAATC | | | |
| **Class II hydrophobin** | | |  | | |
| CsMHP1_QF | | CCGTCATCGCCATTCCC | | | |
| CsMHP1_QR | | GGAGTCTCGCAGAGGACA | | | |
